# UNRAVEL: big data analytics research data platform to improve care of patients with cardiomyopathies using routine electronic health records and standardised biobanking

**DOI:** 10.1007/s12471-019-1288-4

**Published:** 2019-05-27

**Authors:** A. Sammani, M. Jansen, M. Linschoten, A. Bagheri, N. de Jonge, H. Kirkels, L. W. van Laake, A. Vink, J. P. van Tintelen, D. Dooijes, A. S. J. M. te Riele, M. Harakalova, A. F. Baas, F. W. Asselbergs

**Affiliations:** 1 Department of Cardiology, Division Heart & Lungs, University Medical Centre Utrecht, University of Utrecht, Utrecht, The Netherlands; 2 Department of Genetics, Division Laboratories, Pharmacy and Biomedical Genetics, University Medical Centre Utrecht, University of Utrecht, Utrecht, The Netherlands; 3 Department of Methodology and Statistics, Faculty of Social Sciences, University of Utrecht, Utrecht, The Netherlands; 4 Department of Pathology, Division of Pathology, University Medical Centre Utrecht, University of Utrecht, Utrecht, The Netherlands; 5 Institute of Cardiovascular Science, Faculty of Population Health Sciences, University College London, London, UK; 6 Health Data Research UK London and Institute of Health Informatics, University College London, London, UK

**Keywords:** Big data analytics, Biobanking, Cardiomyopathy, Electronic health record, Machine learning, Research data platform

## Abstract

**Introduction:**

Despite major advances in our understanding of genetic cardiomyopathies, they remain the leading cause of premature sudden cardiac death and end-stage heart failure in persons under the age of 60 years. Integrated research databases based on a large number of patients may provide a scaffold for future research. Using routine electronic health records and standardised biobanking, big data analysis on a larger number of patients and investigations are possible. In this article, we describe the UNRAVEL research data platform embedded in routine practice to facilitate research in genetic cardiomyopathies.

**Design:**

Eligible participants with proven or suspected cardiac disease and their relatives are asked for permission to use their data and to draw blood for biobanking. Routinely collected clinical data are included in a research database by weekly extraction. A text-mining tool has been developed to enrich UNRAVEL with unstructured data in clinical notes.

**Preliminary results:**

Thus far, 828 individuals with a median age of 57 years have been included, 58% of whom are male. All data are captured in a temporal sequence amounting to a total of 18,565 electrocardiograms, 3619 echocardiograms, data from over 20,000 radiological examinations and 650,000 individual laboratory measurements.

**Conclusion:**

Integration of routine electronic health care in a research data platform allows efficient data collection, including all investigations in chronological sequence. Trials embedded in the electronic health record are now possible, providing cost-effective ways to answer clinical questions. We explicitly welcome national and international collaboration and have provided our protocols and other materials on www.unravelrdp.nl.

## Introduction

Cardiomyopathies (CMPs) are internationally defined as heart diseases with structurally and functionally abnormal myocardium not explained by coronary artery disease, hypertension or valvular heart disease [1, 2]. Many CMP patients have a familial history of disease, which typically follows an autosomal dominant inheritance pattern. In the Netherlands, it is estimated that 1 in 200 individuals carry a genetic predisposition for a CMP [3–5]. However, penetrance is incomplete and clinical expression of CMPs is heterogeneous, ranging from overt heart failure and lethal arrhythmias to being asymptomatic [2, 6]. Despite major advances in our understanding of the genetics of these diseases, our knowledge of the pathophysiological substrate of CMPs is limited, and CMPs remain a leading cause of premature sudden cardiac death and end-stage heart failure in persons below the age of 60 years [7].

By integrating electronic health records (EHRs) with research data platforms (RDPs), new insights into disease penetrance, risk assessment and disease pathophysiology can be obtained. In their current format, EHRs comprise both structured and unstructured electronic data that have been gathered, captured and assessed during routine clinical care [8]. Major opportunities lie in the standardisation of unstructured data, such as clinical notes and investigations [8–10]. Integrating these data with other data sources, including outcome registries, imaging, wearables and research measurements (‑omics), has the potential of offering higher-resolution data regarding disease epidemiology, onset and progression.

In this article, we present the design of the UNRAVEL RDP, in which a large dataset of CMP patients is enriched by text mining and linked to biomaterials. The UNRAVEL RDP aims to improve the daily care of CMP patients and their family members by (1) providing a standardised database with routine health care data linked to research-generated data that are easily accessible for big data analytics; (2) facilitating harmonisation of data, clinical care protocols and sharing of algorithms on www.unravelrdp.nl; and (3) providing the basis for approaching patients for in-depth biological research through the generation of induced pluripotent stem cells.

## Design

### Ethics and registration

**Fig. 1:**
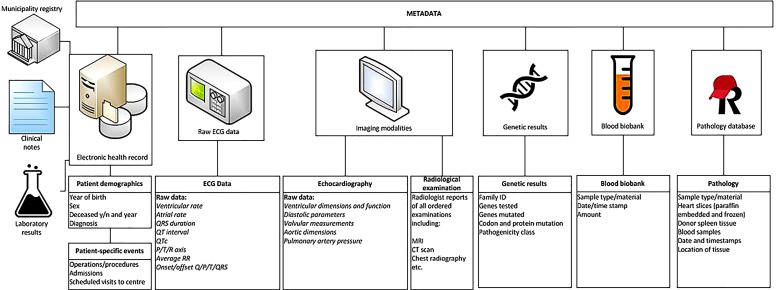
Schematic overview of different types of included data. In short, data on investigations and metadata are automatically extracted after informed consent has been provided. Additionally, patient demographics and specific events, such as date of admission, are included. Information from the municipality registry can be requested concerning, for example, death

The UNRAVEL RDP follows the Code of Conduct and the Use of Data in Health Research and has been approved by the Biobank Board of the Medical Ethics Committee of the University Medical Centre Utrecht (no. 12-387 UNRAVEL Biobank). As a part of UNRAVEL, the use of already existing text files (e.g. clinical notes) is exempt from the Medical Research Involving Human Subjects Act (WMO) as per judgement of the Medical Ethics Committee (Text mining in cardiovascular notes*, *18/446, Utrecht, the Netherlands). Eligible patients (see below) are asked to provide written informed consent for use of their clinical data and previously stored material. Consent is required prior to using the clinical (meta) data. In addition, consent is requested to draw blood via venepuncture during routine investigations, to minimise the impact on the patient, and to request information from other medical centres and municipality registries. For additional stem-cell-related research, an informed consent form has been developed and approved by the Medical Ethics Committee. After inclusion, patients are registered as UNRAVEL enrolees in the EHR, and all their clinical data are automatically collected in the RDP (Fig. 1). Data governance is secured by a data management plan. More information on protocols, data governance and informed consent is provided on www.unravelrdp.nl.

### Study population

Eligible participants are individuals with proven or suspected genetic cardiac disease, and their relatives. UNRAVEL also includes family members who are not mutation carriers or show no signs of disease; these serve as healthy controls. Participants must be able to provide written informed consent and be at least 18 years of age.

In order to minimise selection bias, patients and relatives from both in- and outpatient clinics are prospectively screened and asked to participate. If a participant is deemed eligible after discharge, the patient is contacted by the managing physician by mail and/or phone to retrospectively request consent. Additionally, previously eligible individuals were retrospectively identified and asked to participate using registered diagnoses in the EHR and a database of all CMP patients who visited the outpatient clinic of a clinical geneticist or had DNA analysis performed at the University Medical Centre (UMC) Utrecht.

### Research data platform

**Fig. 2:**
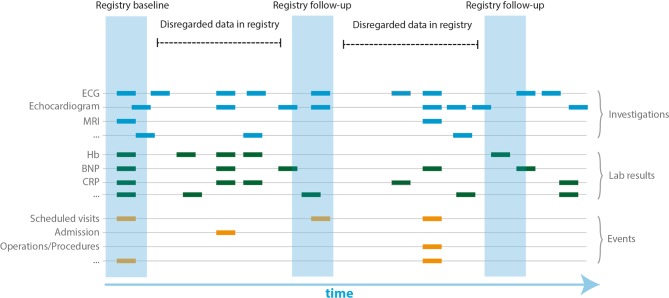
Temporal character of health care data. Schematic overview of a temporal window in which patients visit the centres. In contrast to manually maintained registries where data may be disregarded, the UNRAVEL research data platform includes all (meta)data and investigations. *ECG* electrocardiogram,* MRI* magnetic resonance imaging,* Hb* haemoglobin,* BNP* brain natriuretic peptide, *CRP* C-reactive protein

Consent is required prior to the extraction of data. Based on in-house clinical protocols, phenotyping of participants includes medical history, family history, physical examination, routine laboratory testing, 12-lead electrocardiography, chest radiography, cardiac ultrasonography, computed tomography (CT) and magnetic resonance imaging (MRI). These tests are performed at the discretion of the managing physician and have multiple time points in the EHR (Fig. 2). In contrast to manually maintained registries, all available data are captured. For example, during a visit to the in-patient clinics several electrocardiograms (ECGs) can be produced per day. Not all data might be entered into manually maintained registries, since this is a meticulous and laborious task.

**Fig. 3:**
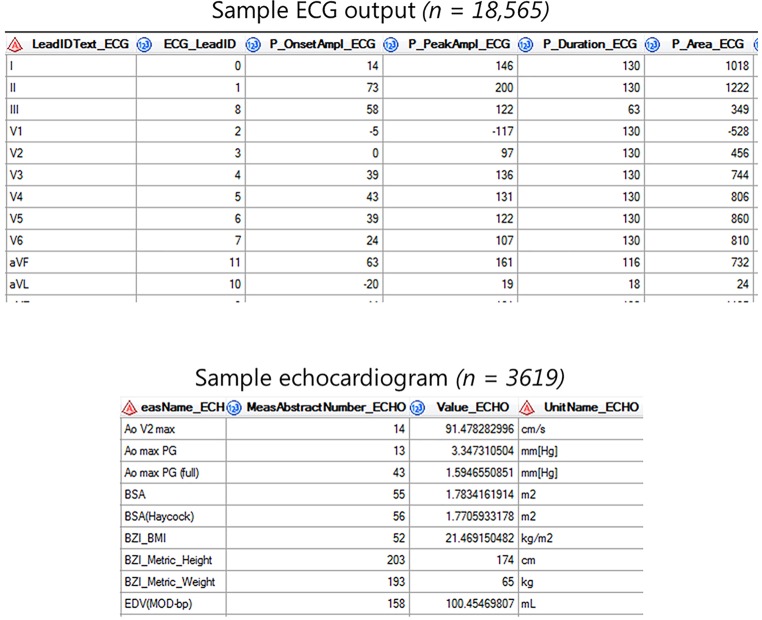
Two data tables from the UNRAVEL research data platform as samples of electrocardiogram and echocardiogram output in SAS enterprise guide. *ECG* electrocardiogram, *ECH* echocardiogram

Raw data is gathered, processed and standardised for all cardiological, electrophysiological, imaging and genetic modalities (Fig. 1). On a weekly basis, these (numeric) data are automatically extracted to the RDP. Metadata is specific information describing the data (such as date of visit, type of ECG, or managing physician) which have been gathered for logistical and administrative purposes. These meta-data harbour valuable information and are also stored in the RDP. Data are viewed, combined, linked to external databases and analysed using query-based searches for data extraction using SAS Enterprise Guide (Fig. 3).

### Outcomes

The UNRAVEL RDP contains multiple outcome measures that can be used for primary or secondary outcome analyses. All-cause death and date of death are extracted from the EHR and retrieved from the municipality registry [11]. Other outcome measures such as diagnoses, date of diagnosis, occurrence of clinical events such as acute heart failure, arrhythmia or hospitalisation, ventricular assist device implantation and clinical interventions, including heart transplantation, can be extracted from the UNRAVEL RDP.

### Text mining

**Fig. 4:**
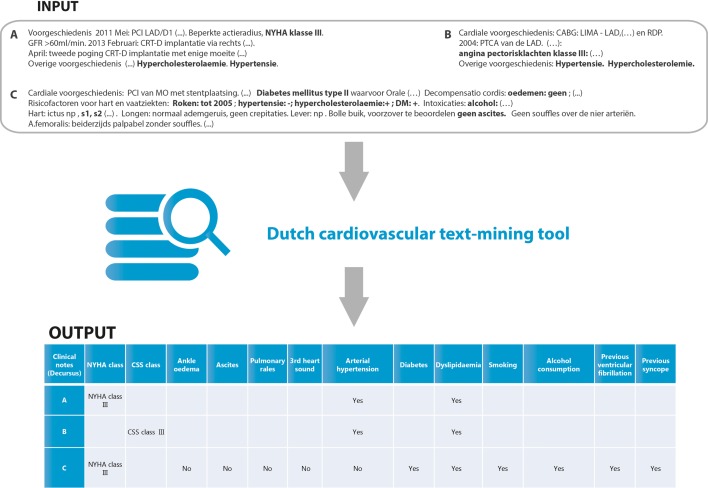
Sample data from the text-mining tool, where based on the clinical notes in the electronic health records (*DECURSUS)* an output file is created with different standardised variables, such as arterial hypertension, diabetes and dyslipidaemia. Variables are harmonised with the German TORCH registry, but can be changed as deemed necessary. The application is written for Dutch cardiovascular notes

The UNRAVEL RDP includes all structured data from the EHR. However, some data remain unstructured, such as free text. These texts might harbour valuable variables to extract, such as New York Heart Association (NYHA) class or other clinical symptoms. To enrich the UNRAVEL RDP with these unstructured data from clinical notes, a text-mining prototype tool was developed. In short, we defined pre-set variables for the tool to extract from clinical notes, e.g. NYHA classification and cardiovascular risk factors such as diabetes, hypercholesterolaemia and hypertension. The pre-set variables are now in accordance with the variables in the TORCH registry but can be defined at the discretion of the researcher [12]. The algorithm and further explanation are provided open source on www.unravelrdp.nl. Since the tool is under development, it should only be used with caution and under the supervision of medical and text-mining experts until further evaluation. A sample output of this automated tool is presented in Fig. 4. Future perspectives include the use of natural language processing for automated standardised diagnosis registration from clinical notes based on the International Classification of Disease (ICD) 10 classification mapped to the diagnosis thesaurus and reimbursement codes set by the project group “DHD diagnosis thesaurus-DBC-ICD 10” of the Dutch Society of Cardiology [13]. Data standardisation will be harmonised with the OMOP Common Data Model to allow for systematic analysis of disparate observational databases [14].

### Blood biobank

All patients are asked concerning the collection of biomaterials for the UNRAVEL Blood Biobank. The exact laboratory protocol is available on www.unravelrdp.nl. In short, the standardised biobank protocol consists of one 10 ml serum, one 4.5 ml citrate, one 2 ml ethylenediaminetetraacetic acid (EDTA), one 10 ml EDTA and one 10 ml Na-heparin blood collection tube. These are processed and aliquoted to two vials of 0.5 ml whole blood from EDTA tubes, four vials of 0.5 ml plasma from citrate tubes, six vials of 0.5 ml plasma from EDTA and heparin tubes and six vials of 0.5 ml serum. All samples are stored at −80 °C. Availability, type and storage of material are linked to the RDP for easy accessibility.

### Cardiac tissue database

Cardiac tissue of patients that have received a left ventricular assist device or undergone heart transplantation, and received donor spleen tissue during heart transplantation are routinely stored by the Department of Pathology. Samples are paraffin embedded and frozen at −80 °C. All samples are stored according to the protocol available on www.unravelrdp.nl, and explanted hearts are divided into slices and cubes accordingly. The registration of these samples is performed using an electronic case registration form in Redcap in the cardiac tissue database which is linked to the UNRAVEL RDP. Further information can be found on www.unravelrdp.nl.

## Preliminary results

An overview of the preliminary results is provided in Tab. 1. By October 2018, 1928 individuals had been asked to participate in the UNRAVEL RDP. Of these, 828 individuals provided consent, of which 58% are male. Median current age is 57 years (interquartile range (IQR) 45–67). Overall, the available data comprises 18,565 ECGs with a median of 74 per patient (IQR 32–105), 3619 different echocardiograms with a median of 12 per patient (IQR 5–18), data from over 20,000 radiological examinations including 389 cardiac MRI scans and 650,000 individual laboratory results. Data from other non-cardiac examinations, e.g. orthopaedic MRI or endoscopy, are also available. In 356 participants, a diagnosis of heart failure had been registered according to the diagnosis thesaurus described earlier: 222 have dilated CMP, 38 hypertrophic CMP. Blood from 267 patients has thus far been stored in the biobank according to protocol. To date, 323 mutations have been identified, primarily in *PKP2 *(23%), *PLN *(17%) and *TTN *(13%).

**Table 1:** Clinical characteristics and available tests of 828 patients included in UNRAVEL. Data are presented as number (median, IQR)

Male	480 (58%)
Median age	57 years (IQR 45–67)
**Diagnosis as registered in EHR**	
Heart failure	356
DCMP	222
HCMP	38
Cardiooncology	95
Not specified	308
Cardiogenetic screening	165
Cardiac ultrasound images	3619 (12, IQR 5–18)
Electrocardiograms	18,565 (74, IQR 32–105*)*
**Radiological examinations**	20,318
Chest radiography	512
CT thorax	274 (7, IQR 3–15*)*
MRI cardiac	389 (2, IQR 1–3*)*
Laboratory tests	650,000
Biobanking	267
**Device therapy**	241
LVAD	46
ICD/CRT	195
Heart transplantation	72
**Genes mutated**	323
*PKP2*	76
*PLN*	54
*TTN*	41
*MYBPC3*	38
*MYH7*	13
*LMNA*	10
Other	91

*IQR* interquartile range, *EHR* electronic health record, *DCMP* dilated cardiomyopathy, *HCMP* hypertrophic cardiomyopathy, *CT* computed tomography, *MRI* magnetic resonance imaging, *LVAD* left ventricular assist device, *ICD* internal cardiac defibrillator, *CRT* cardiac resynchronisation therapy

*MRI cardiac* includes both MRI cardiac and stress MRI (adenosine/dobutamine). Radiological examinations include all examinations performed in-house, e.g. chest, abdominal, thyroid radiography etc

## Discussion

There is still limited knowledge on the aetiology, diagnostic performance of clinical investigations and disease modifiers in CMPs, complicating the clinical care of these patients [2, 6, 7]. Research databases based on large numbers of patients provide the infrastructure for new insights into these diseases. To date, patient registries have typically often had fixed time points at which data are manually inputted, data entry is at the discretion of the researcher and a vast amount of (meta)data gathered during routine clinical care is inherently disregarded. The current advanced EHR systems provide exciting opportunities to access all data gathered in routine clinical care which can be linked to research data. The resulting datasets will have larger resolution and may provide new insights into disease penetrance, risk assessment and disease pathophysiology [8, 15]. The UNRAVEL RDP incorporates these large automated and standardised datasets of CMP patients, enriched with language processing and text retrieval. Advantages include (1) automation and efficiency, (2) featuring temporal or sequential data, (3) allowing for EHR-embedded trials and (4) mining unstructured data using text analysis.

EHR data are extracted and standardised in the UNRAVEL RDP, which has thus far led to a dataset comprising 828 patients with a total of 18,565 ECGs, 3619 echocardiograms, 389 cardiac MRI scans and 323 patients with mutated genes (Tab. 1). The RDP automatically provides these raw (meta)data. This obviates the laborious need for manually maintained registries, saving the precious time of (medical) experts and reducing transcription errors. Furthermore, since outcomes such as admission, heart transplantation and (cardiac) death are automatically extracted from the EHR, obtaining follow-up will be less time-consuming, thereby reducing costs [11].

With the RDP, these data can be integrated into a detailed longitudinal picture of the clinical course of a patient, a “human phenome sequence” [8]. In previous studies, (semi-)supervised and unsupervised machine learning on linked EHR data was able to solve problems in prediction and pattern recognition [8, 16, 17]. However, routine clinical records can be sparsely filled and (ontological) definitions of disease may differ over time. To counter these issues, a semi-supervised machine learning method has been proposed by Beaulieu-Jones et al. [18] to analyse these high-dimensional EHR data, constructing phenotypes based on unsupervised learning, then clustering these patients in sub-phenotypes and performing survival analyses. Furthermore, large datasets such as the UNRAVEL RDP are prone to generate associations with uncertain causal relevance. To address causality, the addition of our stem-cell informed consent serves as a stepping stone for functional follow-up studies using induced pluripotent stem cells. Additional statistical frameworks such as instrumental variables and Mendelian randomisation, or further research in randomised clinical trials may also provide further support to observed associations [19].

To embed clinical trials, data in the UNRAVEL RDP can be used for trial feasibility, patient recruitment, but also for remote data monitoring, potentially reducing clinical trial costs and selection bias (pragmatic trials). Using the UNRAVEL RDP, it is possible to perform interventions and measure outcomes during routine health care, ranging from life-style interventions to logistical questions on how often a patient should be followed up. EHR can be an alternative to electronic case registration forms providing data is consistently collected in routine clinical care, including data on (adverse) events [20].

Structured EHR data such as encoded diagnosis and cardiac ultrasound are the easiest data sources to process, but advances in text mining have made it possible to also use unstructured clinical data, such as patient medical histories, discharge summaries and clinical notes [10, 14]. Using a text-retrieval algorithm, we have developed a tool to extract standardised data from clinical notes. This tool is, however, still under development and was implemented on clinical notes from the Department of Cardiology at the UMC Utrecht. Therefore, the tool should be used with caution and under the supervision of a medical expert in other centres.

EHR data that are subjected to robust pre-processing and cleaning have been shown to offer a common scaffold upon which research questions can be built and linked to datasets, enabling new areas of research [9, 21]. With these “big” EHR data, however, great challenges and responsibilities arise: data governance, data access, public trust, definitions of disease and development of replicable scientific tools. Furthermore, these large datasets are prone to generating associations with great uncertainty regarding causality. Therefore, analysis of data and interpretation must be performed by a multidisciplinary team including medical experts, epidemiologists and data scientists. Only if the data are understood and carefully evaluated can new models explaining onset and progression of disease be developed [8].

In conclusion, the UNRAVEL RDP is an enriched data platform for CMPs that combines EHR data with a standardised blood biobank and text-mining tools. This integration of EHR data into the RDP allows novel analysis of the onset and progression of disease and can embed performance measures in clinical practice. Laboratory protocols, informed consent forms and algorithms are available on www.unravelrdp.nl. Protocols have been shared thus far with the University Medical Centre Groningen, Amsterdam University Medical Centre and Bergman Clinics, and we explicitly welcome national and international cooperation with the UNRAVEL team to harmonise protocols.
